# Supervised Physical Training Enhances Muscle Strength but Not Muscle Mass in Prostate Cancer Patients Undergoing Androgen Deprivation Therapy: A Systematic Review and Meta-Analysis

**DOI:** 10.3389/fphys.2019.00843

**Published:** 2019-07-03

**Authors:** Ziyuan Chen, Yuan Zhang, Chunyan Lu, Hao Zeng, Moritz Schumann, Sulin Cheng

**Affiliations:** ^1^Department of Physical Education, Exercise, Health and Technology Centre, Shanghai Jiao Tong University, Shanghai, China; ^2^The Key Laboratory of Systems Biomedicine, Ministry of Education, and The Exercise Translational Medicine Centre, Shanghai Center for Systems Biomedicine, Shanghai Jiao Tong University, Shanghai, China; ^3^Department of Endocrinology, West China Hospital, Sichuan University, Chengdu, China; ^4^Department of Urology, West China Hospital, Sichuan University, Chengdu, China; ^5^Department of Molecular and Cellular Sport Medicine, Institute of Cardiovascular Research and Sport Medicine, German Sport University, Cologne, Germany; ^6^Faculty of Sport and Health Sciences, University of Jyväskylä, Jyväskylä, Finland

**Keywords:** ADT, androgen suppression, lean mass, exercise medicine, strength training, exercise oncology

## Abstract

**Introduction:** Androgen deprivation therapy (ADT) is considered the basic treatment for advanced prostate cancer, but it is highly associated with detrimental changes in muscle mass and muscle strength. The aim of this meta-analysis was to investigate the effects of supervised physical training on lean mass and muscle strength in prostate cancer patients undergoing ADT.

**Methods:** A systematic literature search was performed using MEDLINE, Embase, and ScienceDirect until October 2018. Only studies that examined both muscle mass and strength in prostate cancer patients undergoing ADT were included. Outcomes of interest were changes in lean body mass (surrogate for muscle mass) as well as upper and lower body muscle strength. The meta-analysis was performed with fixed-effects models to calculate mean differences between intervention and no-training control groups.

**Results:** We identified 8,521 publications through the search of the following key words: prostate cancer, prostate tumor, prostate carcinoma, prostate neoplasm, exercise, and training. Out of these studies, seven randomized controlled trials met the inclusion criteria and where included in the analysis. No significant mean differences for changes in lean mass were observed between the intervention and control groups (0.49 kg, 95% CI: −0.76, 1.74; *P* = 0.44). In contrast, the mean difference for muscle strength was significant both in chest (3.15 kg, 95% CI: 2.46, 3.83; *P* < 0.001) and in leg press (27.46 kg, 95% CI: 15.05, 39.87; *p* < 0.001).

**Conclusion:** This meta-analysis provides evidence that low- to moderate-intensity resistance and aerobic training is effective for increasing muscle strength but may not be sufficient to affect muscle mass in prostate cancer patients undergoing ADT. The underlying mechanisms for this maladaptation may in part be explained by an insufficient stimulus induced by the training regimens as well as a delayed initiation of training in relation to the start of ADT. When interpreting the present findings, one should bear in mind that the overall number of studies included in this review was rather low, emphasizing the need for further studies in this field.

## Introduction

Prostate cancer has become the second most common cancer in developed countries, affecting especially older men (American Cancer Society, 2013). Because testosterone exacerbates prostate cancer, androgen deprivation therapy (ADT) is considered preferentially as the basic treatment (Pagliarulo et al., 2012; Crawford and Moul, 2015). Thus, over one million patients received or are currently receiving ADT in the United States (Smith, 2007; DeSantis et al., 2014; Tsai et al., 2015; Pagliarulo, 2018), either through surgery (bilateral orchiectomy) or medication (i.e., by gonadotropin-releasing hormone [GnRH] agonists or GnRH antagonists; Ahmadi and Daneshmand, 2014).

Anabolic steroids such as testosterone are known to play a crucial role in muscle growth both in healthy and in diseased populations (Shabsigh et al., 2009; Bandak et al., 2016). Consequently, significant reductions of circulating testosterone concentrations induced by ADT typically lead to serious adverse events. These include but are not limited to a loss of lean mass (Vermeulen et al., 1999; Galvão et al., 2008), bone mineral density (Galvão et al., 2008), and muscle strength (Araujo et al., 2007), with concomitant increases in fat mass (Vermeulen et al., 1999). These unfavorable changes may, in turn, have an adverse impact on overall quality of life and increase the risk of falls and hip fractures (Shahinian et al., 2005).

Previous studies have well-documented that regular physical exercise can ameliorate many of the common adverse effects of ADT (Segal et al., 2003; Galvão et al., 2010; Cormie et al., 2013). Resistance and/or aerobic training have been shown to improve muscle strength and aerobic capacity, reduce fatigue, and improve overall quality of life (Keogh and MacLeod, 2012). However, few studies have focused on the effects of exercise on muscular strength and lean body mass in prostate cancer patients undergoing ADT (Gardner et al., 2014). Recent studies have shown that muscle mass is an important predictor of overall survival in patients with various cancer entities (Beuran et al., 2018; Dolan et al., 2018; Limpawattana et al., 2018), including prostate cancer (Cushen et al., 2016).

In light of suppressed testosterone concentrations, it remains questionable whether physical exercise provides a sufficient stimulus to increase muscle mass in men receiving ADT. Although anabolic steroids certainly play a crucial role in protein synthesis, muscle growth might also be induced by other means, such as other growth factors (e.g., insulin-like growth-factor I) as well as amino acid or mechanical signaling (Hoppeler, 2016; Wackerhage et al., 2019). Theoretically, this also provides potential for patients treated with ADT to gain muscle mass, especially by exercise protocols typically recommended to target at muscle hypertrophy (i.e., 8–12 repetitions at 70–85% of maximal strength Kraemer and Ratamess, 2004).

This systematic review and meta-analysis primarily aimed to investigate whether supervised physical training significantly increases lean body mass in prostate cancer patients treated with ADT. Moreover, we investigated whether possible changes in lean mass were translated into improvements in muscle strength. To ensure accuracy of our findings, only randomized-controlled trials with objective measures of lean mass, such as a dual-energy X-ray absorptiometry (DXA) were considered. Therefore, our homogeneous analysis differs from a recent systematic review which was not limited to studies with objective measures for muscle mass (Gardner et al., 2014), as well as a recent meta-analysis which included prostate cancer patients who were not required to be treated with ADT (Keilani et al., 2017).

## Methods

A systematic literature search was conducted in accordance with the Preferred Reporting Items for Systematic Reviews and Meta-Analyses (PRISMA) (Liberati et al., 2009) and was registered at the international database of prospectively registered systematic reviews in health and social care (PROSPERO: CRD42018094240).

The search was performed using MEDLINE, Embase, and ScienceDirect. Databases were searched from their inception until October 2018 by two independent researchers (CZY, ZY). Search terms related to prostate cancer (e.g., prostate tumor, prostate carcinoma, and prostate neoplasm) and exercise (exercise, training) were used (Table 1). The search process included removing duplicates and screening titles, abstracts, and eligible full texts. The reference lists of included studies were also checked for additionally relevant studies.

**Table 1:** Search strategy for each data base.

**Databases**	**Search strategy**
MEDLINE	(Prostate cancer^*^[Title/Abstract])OR (Prostate tumor^*^[Title/Abstract])OR (Prostate carcinoma^*^[Title/Abstract])OR (Prostate tumor^*^[Title/Abstract])OR (Prostate neoplasm) AND (exercise^*^[Title/Abstract]) OR (Training [Title/Abstract])
Embase	(“prostate cancer”:ab,ti OR “prostate tumor”:ab,ti OR “prostate carcinoma”:ab,ti OR “prostate tumor”:ab,ti OR “prostate neoplasms”:ab,ti) AND (“exercise”:ab,ti OR “training”:ab,ti)
ScienceDirect	(“Prostate cancer” OR “Prostate tumor” OR “Prostate carcinoma” OR “Prostate tumor” OR “Prostate neoplasm”) AND (“exercise” OR “Training”)

### Eligibility Criteria

In line with the aim of this meta-analysis, only studies published in peer-reviewed scientific journals in English language were included. The detailed inclusion criteria followed the PICO (participants, interventions, comparators, outcomes, and study design; Liberati et al., 2009). The population of this review included patients currently receiving any form of ADT (i.e., bilateral orchiectomy or hormone antagonists), and studies were required to include at least one group performing supervised exercise, as well as a no-training control group. All studies fulfilling these criteria regardless of age, prostate tumor stage, and other concomitant treatments (e.g., radiology, chemotherapy, and prostatectomy) were deemed eligible. The outcomes of interest included measures of both lean mass and muscle strength. Thus, only studies reporting data of lean mass and muscle strength both before and after the trial were eligible. In addition, only randomized-controlled trials (RCT) were considered appropriate. The detailed inclusion criteria were as follows: (1) The duration of the exercise intervention was longer than 3 months; (2) An objective measure of lean mass (e.g., dual-energy X-ray absorptiometry; Pietrobelli et al., 2001) and muscle strength (e.g., one repetition maximum test) was implemented; (3) The intervention included exercise training only (i.e., no nutritional supplementation, or combined exercise and other treatments); (4) A control group with no supervised physical training was included, while placebo control conditions including e.g., psychosocial support as well as stretching and relaxation were allowed. Studies that did not meet all of these criteria were excluded.

### Data Extraction

Basic information on the sample, the type of intervention, and relevant study outcomes were extracted from each original study and summarized into draft forms. Points of disagreement were discussed first and then judged by a third author (MS). In detail, the following data was extracted from each eligible study: (1) the general characteristics (e.g., the last name of first author, year of publication, aim of the study and outcomes); (2) participant information (e.g., sample size and age); (3) intervention data for the exercise and control groups (e.g., intervention duration, types of interventions); (4) specific outcomes (i.e., muscle mass and muscle strength). Due to the use of different terms to describe changes in body composition, the following terms were summarized as muscle mass/lean body mass: total-body lean mass, whole-body lean mass, total lean mass, lean tissue mass, and fat-free lean mass. In studies in which the prescribed intervention continued after the supervised training period, for example, by home-based exercise, only the duration of supervised training was included in the analysis.

### Data Analysis

The meta-analysis was conducted by Revman 5.3 (version 5.3, the Nordic Cochrane Centre, Copenhagen, Denmark). Mean changes and corresponding standard deviations (SD) from baseline to the endpoint of each study were calculated. Taking into account the same outcome and unit of measurement, study results were pooled by using the mean difference (MD) for lean body mass and muscle strength, according to Cohen (1988). In addition, a fixed-effects model was used for the same measurement of outcomes and similar intervention duration. Statistical heterogeneity was assessed by a chi-squared test and *I*^2^. The *I*^2^ values >50% indicated a large heterogeneity. Publication bias was assessed by funnel plots. All data was presented with 95% confidence intervals (CI).

### Risk of Bias Assessment

The Cochrane Collaboration's risk of bias assessment tool (Higgins et al., 2011) was used to evaluate the internal validity of the included randomized controlled trials (RCTs). Independently, two authors (CZY, ZY) examined the studies of interest for the following sources of bias: selection (sequence generation and allocation concealment), performance (blinding of participants/personnel), detection (blinding outcome assessors), attrition (incomplete outcome data), reporting (selective reporting), and other potential bias (e.g., recall bias). Although blinding is not feasible in exercise interventions, this quality criterion was still assessed for integrity and in agreement with other systematic reviews in the field.

## Results

### Study Characteristics

**Figure 1:**
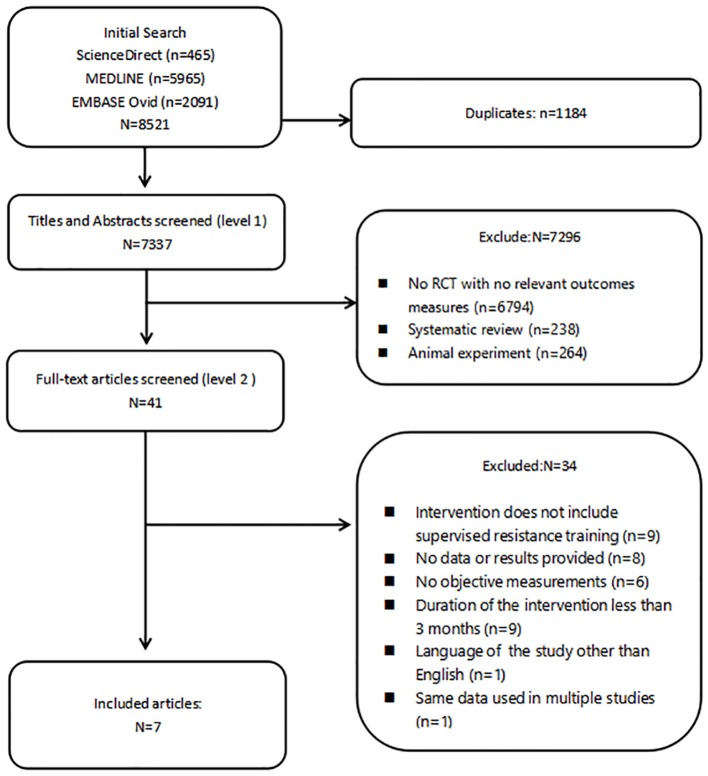
Screening chart.

The results of the literature search are summarized in Figure 1. Eight thousand five hundred and twenty-one records were retrieved. After removing duplicates, 7,337 eligible articles remained for further analysis. Following screening of titles and abstracts, 41 records were deemed relevant to the theme and retrieved for full-text review. Finally, only seven studies remained for the present meta-analysis.

All seven studies compared an exercise intervention with a no-training control group, receiving usual care (Galvão et al., 2010, 2014; Nilsen et al., 2015), support information (Galvão et al., 2014; Taaffe et al., 2018), stretching (Winters-Stone et al., 2015), or maintenance of their normal physical activity and dietary routine (Wall et al., 2017). Out of these included studies, in two trials solely supervised whole-body resistance training was performed by the intervention group (Nilsen et al., 2015; Winters-Stone et al., 2015), whereas in the five remaining trials, training consisted of a combination of resistance and aerobic training (Galvão et al., 2010, 2014; Cormie et al., 2015a; Wall et al., 2017; Taaffe et al., 2018). Dropout rates ranged from 3 to 21% and were mainly related to non–exercise related adverse events as well as personal reasons, such as a loss of interest (Galvão et al., 2010, 2014; Winters-Stone et al., 2015; Wall et al., 2017; Taaffe et al., 2018), health-related reasons (Nilsen et al., 2015), and travel constraints (Cormie et al., 2015a).

The meta-analysis was carried out with a total of 468 patients at a mean age of 69.7 (SD = 7.3) years. Six studies (Galvão et al., 2010, 2014; Cormie et al., 2015a; Winters-Stone et al., 2015; Wall et al., 2017; Taaffe et al., 2018) excluded patients with bone metastatic disease, and one study (Nilsen et al., 2015) excluded patients using osteoporosis medication. The ADT treatment differed between studies, ranging from 6 days to 39 months. Furthermore, the length of the exercise interventions varied across studies, ranging from 3 to 12 months. The majority of studies used a training frequency of two sessions per week (Galvão et al., 2010, 2014; Cormie et al., 2015a; Winters-Stone et al., 2015; Wall et al., 2017; Taaffe et al., 2018), with the exception of one study (Nilsen et al., 2015) in which three weekly sessions were performed. The intensity of aerobic exercise among the studies ranged from 65 to 90% of maximum heart rate, whereas strength training was mainly performed with a 6–12 repetition maximum (RM; i.e., the maximal weight that can be lifted 6–12 times). The training characteristics of included studies are summarized in Table 2.

**Table 2:** Characteristic information of the included studies.

**References**	**Number of patients**	**ADT duration**	**Intervention**	**Supervised exercise duration**	**Frequency (hour)**	**Intensity**	**Drop-out rate**	**Findings**	***P-value***
Taaffe et al., 2018	T: *N* = 57 EX: *N* = 28 CON: *N* = 29	6 months: EX: *n* = 12 CON: *n* = 17 18 months: EX: *n* = 16 CON: *n* = 12	EX: combined progressive supervised resistance (whole-body training) and aerobic exercise CON: receiving physical activity advice supported with printed material	6 months	Twice per week (1 h)	Resistance training with loading progressing from 6 to 12 repetition maximum (RM) for two to four sets per exercise. The intensity of aerobic exercise was set at 70–85% of maximum heart rate and perceived exertion at 11–13 on the Borg Rating of Perceived Exertion Scale (6–20 points).	Unclear	EX: Lean body mass: +0.1 KG (SD change ±6.2) Chest press: +2.5 KG (SD change ±9.2) Leg press: +23.7 KG (SD change±37.1) CON: Lean body mass: −0.1 KG (SD change ±6.5) Chest press: −0.6 KG (SD change ±10.4) 3. Leg press: +9.6 KG (SD change±52.2)	Between group: not reported Within group: not reported
Wall et al., 2017	T: *N* = 97 EX: *N* = 50 CON: *N* = 47	ADT time, months median EX: 3.0 (2.0–4.0) CON: 2.0 (2.0–3.5)	EX: resistance (whole-body training) and aerobic training CON: maintaining normal physical activity and dietary routine	6 months	Twice per week (1 h)	The intensity of resistance exercise was 4-wk cycle, 6–12 RM (e.g., the maximal weight that can be lifted 6 to 12 times) using one to four sets per exercise. The intensity of aerobic exercise was 70–90% of each participant's measured heart rate at VO2 max.	21%	EX: Lean body mass: +0.7 KG (SD change ±8.6) Body mass: +0.8 KG (SD change±16.7) CON: Lean body mass: –0.1 KG (SD change ±6.0) Body mass: +1.9 KG (SD change±12.3)	*Between groups:* Lean body mass: 0.015 Body mass:0.055 Within group: not reported Within group: not reported
Nilsen et al., 2015	T: *N* = 58 EX: *N* = 28 CON: *N* = 30	Average ADT duration: 9 months	EX: high-load strength training (whole-body training) program CON: usual care	16 weeks	Three sessions per week	Exercise with low resistance corresponding to 40–50% of one RM. Training volume through the intervention period: from one to three sets of 10 RM on Mondays, and from two to three sets of 6 RM on Fridays. A sub maximal session was carried out on Wednesdays, with 10 repetitions and 80–90% of 10 RM in 2–3 sets.	15%	EX: Lean body mass: +0.5 KG (SD change ±7.2) Body mass: +0.4 KG (SD change ±12.4) Chest press: +5 KG (SD change ±12) Leg press: +44 KG (SD change±54.5) CON: Lean body mass: ±0 KG (SD change ±6.7) Body mass: +0.1 KG (SD change ±12.5) Chest press: ±0 KG (SD change±11) 4. Leg press: ±0 KG (SD change±42)	*Between groups:* Lean body mass: 0.175 Body mass: 0.509 Chest press: <0.001 Leg press: <0.001 Within group: not reported
Winters-Stone et al., 2015	T: *N* = 58 EX: *N* = 28 CON: *N* = 30	Average time undergoing ADT (months) EX:39 CON:28.5	EX: resistance (whole-body training) and impact training exercise CON: stretching placebo control	12 months	Twice per week (1 h)	Resistance training used free weights for 1–3 sets per exercise at a weight that could be lifted for 8–12 repetitions (about 60–80% of one repetition maximum [1RM]).	13% drop out	EX: Lean body mass: ±0 KG (SD change ±9.3) Body mass: –0.4 KG (SD change±15) CON: Lean body mass: –0.3 KG (SD change±6.6) 2. Body mass: +0.6 (SD change±13.8)	Between group: not reported Within group: not reported
Cormie et al., 2015a	T: *N* = 63 EX: *N* = 32 CON: *N* = 31	Average time since ADT injection (days) EX:6.2 CON: 5.6	EX: moderate–high intensity aerobic and resistance (whole-body training) exercise CON: usual care	3 months	Twice per week (1 h)	The intensity of resistance exercise was manipulated from 6–12 repetition maximum using 1–4 sets per exercise. The intensity of aerobic exercise was set at approximately 70%−85% of estimated maximum heart rate.	12%	EX: Lean body mass: –0.6 KG (SD change ±6.4) Body mass: –1.2 KG (SD change ±13.3) Chest press: +2.4 KG (SD change ±13.0) Leg press: +23.6 KG (SD change±51.5) CON: Lean body mass: –1.4 KG (SD change ±6.5) Body mass: –0.6 (SD change ±10.3) Chest press: –3 KG (SD change±15.8) Leg press: –1.9 KG (SD change±48.3)	Between group: not reported Within group: EX: Lean body mass: 0.168 Body mass: 0.170 Chest press: <0.001 Leg press:0.038 CON: Lean body mass: <0.001 Body mass: 0.061 Chest press: 0.012 Leg press:0.369
Galvão et al., 2014	T: *N* = 100 EX: *N* = 50 CON: *N* = 50	Average previous ADT duration (months) EX:12.9 CON:11.0	EX: resistance (whole body training) and aerobic exercise CON: printed educational material	6 months	Twice per week (total time not reported)	The intensity of resistance exercises was set at 6–12 repetition maximum (RM) for two to four sets per exercise. The intensity of aerobic exercise was set at 70–85% maximum heart rate and perceived exertion at 11–13 (6–20 point Borg scale).	13% drop out	EX: Lean body mass: +0.1 KG (SD change ±6.2) Body mass: −0.3 KG (SD change ±12.4) Chest press: +2.6 KG (SD change±1.8) CON: Lean body mass: −0.1KG (SD change ±6.5) Body mass: −0.6KG (SD change ±11.9) Chest press: −0.5KG (SD change ±1.8)	Between group: Lean body mass: 0.290 Body mass: 0.777 Chest press:0.004 Within group: no report
Galvão et al., 2010	T: *N* = 57 EX: *N* = 29 CON: *N* = 28	Average AST time, months EX:18.2 CON:10.1	EX: resistance (whole-body training) and aerobic exercise CON: usual care	12 weeks	Twice per week (total time not reported)	The resistance exercise program was designed to progress from 6–12 repetition maximum (RM) for two to four sets per exercise. The intensity of aerobic exercise was set at 65–80% maximum heart rate and perceived exertion at 11–13 (6–20 point, Borg scale).	3%	EX: Lean body mass: +0.7 KG (SD change ±6.8) Body mass: +0.7 KG (SD change ±10.5) Chest press: +3.8 KG (SD change ±11.3) Leg press: +36.2 KG (SD change±48.6) CON: Lean body mass: ±0 KG (SD change ±9.5) Body mass: ±0 KG (SD change ±14.4) Chest press: +0.5 KG (SD change±12.9) Leg press: +7 KG (SD change±53.7)	Between group: Lean body mass: 0.047 Body mass: 0.163 Chest press: 0.018 Leg press: <0.001 Within group: no report

*In the control group, no supervised physical training was performed. RM, repetition maximum; T, Total; EX, exercise training group; CON, control group; SD, standard deviation*.

### Risk of Bias Assessment

**Figure 2:**
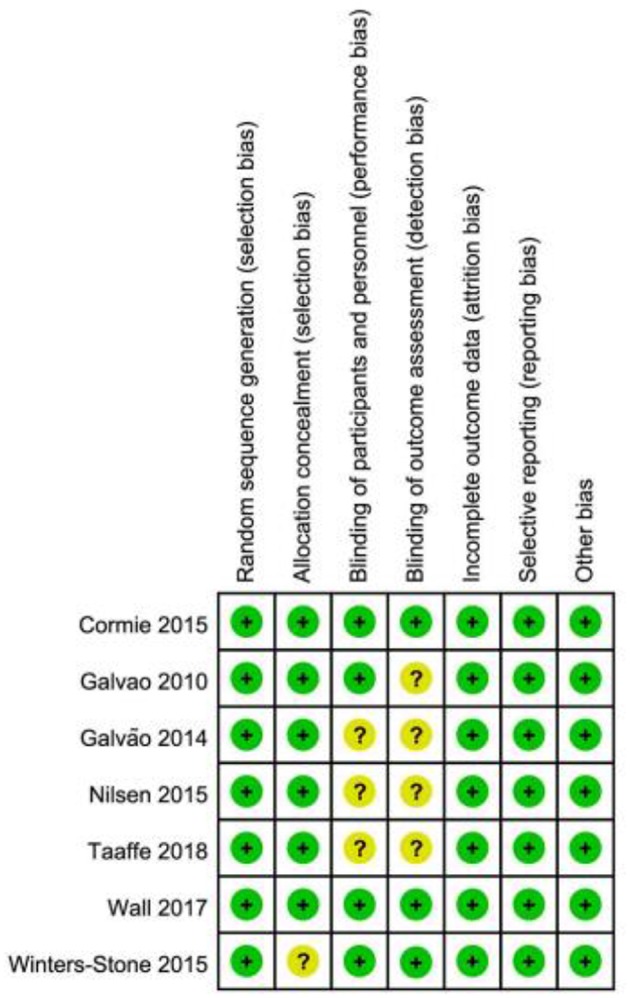
Summary of risk of bias assessment.

Risk of bias was low in two studies (Cormie et al., 2015a; Wall et al., 2017), while it was uncertain the remaining studies (Figure 2; Table 3).

**Table 3:** Summarized risk of bias for all included studies.

**References**	**Results of bias assessment**
Cormie et al., 2015a	Low risk
Galvão et al., 2010	Unclear risk
Galvão et al., 2014	Unclear risk
Nilsen et al., 2015	Unclear risk
Taaffe et al., 2018	Unclear risk
Wall et al., 2017	Low risk
Winters-Stone et al., 2015	Unclear risk

### Changes in Lean Mass

**Figure 3:**
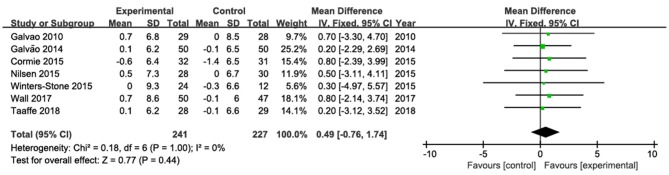
Forest plots for pooled mean differences in lean mass with corresponding 95% CI.

The results of lean mass were pooled from seven studies (Galvão et al., 2010, 2014; Cormie et al., 2015a; Nilsen et al., 2015; Winters-Stone et al., 2015; Wall et al., 2017; Taaffe et al., 2018; Figure 3). No statistically significant change in lean mas was observed. The pooled mean difference in total lean mass was 0.49 kg (95% CI: −0.76, 1.74; *P* = 0.44), with low heterogeneity (*I*^2^ = 0%).

### Changes in Upper- and Lower-Body Muscle Strength

**Figure 4:**
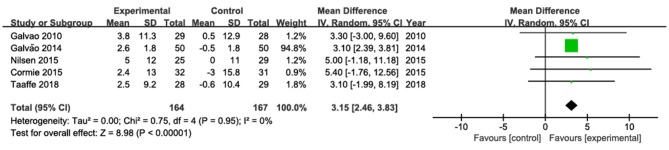
Forest plots for pooled mean differences in chest press strength with corresponding 95% CI.

The results of chest press were pooled from four studies (Galvão et al., 2010, 2014; Nilsen et al., 2015; Taaffe et al., 2018; Figure 4). Among them, only one study (Galvão et al., 2014) showed a statistically significant increase in chest press. The pooled estimate of mean difference in chest press was 3.15 kg (95% CI: 2.46, 3.83; *P* < 0. 001), with low heterogeneity (*I*^2^ = 0).

**Figure 5:**
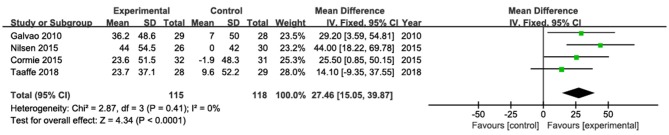
Forest plots for pooled mean differences in leg press strength with corresponding 95% CI.

The results of leg press were pooled from four studies (Galvão et al., 2010; Cormie et al., 2015a; Nilsen et al., 2015; Taaffe et al., 2018; Figure 5). Among them, three studies showed significantly increased leg press strength (Galvão et al., 2010; Cormie et al., 2015a; Nilsen et al., 2015), whereas one study (Taaffe et al., 2018) did not found significant changes after the intervention. The pooled estimate of mean difference in leg press was 27.46 kg (95% CI: 15.05, 39.87; *p* < 0. 001), with low heterogeneity (*I*^2^ = 0).

## Discussion

In this meta-analysis, we examined the effects of supervised exercise on muscle mass and strength in cancer patients undergoing ADT. The main findings indicated that supervised exercise does not statistically affect total lean mass compared to usual care, whereas the pooled mean difference for upper- and lower-body muscle strength was significant in favor of the training interventions.

Over the past decade, evidence of the importance of physical exercise as a supportive therapy for the management of cancer has considerably increased (Bourke et al., 2016). Exercise is known to be safe and feasible for prostate cancer patients (Cormie et al., 2015b) and widely accepted as an effective adjuvant therapy (Richman et al., 2011; Ashcraft et al., 2016; Galvão et al., 2016; Peisch et al., 2017) by ameliorating many of the side effects induced by the medical treatment. Our meta-analysis adds to previous knowledge by highlighting that the positive effects of physical exercise do not necessarily induce changes in lean mass in patients receiving ADT.

The underlying causes for our findings may be manifold. ADT is known to cease testosterone production by the testicles. Testosterone, in turn, is considered one of the most potent androgens, critically controlling muscle protein synthesis (Vermeulen et al., 1999; Vingren et al., 2010). For example, in healthy elderly people, declines in muscle mass (i.e., sarcopenia) are typically associated with reductions in anabolic hormone concentrations (Doherty, 1985; Morley et al., 2001; Shin et al., 2018). Furthermore, previous studies have shown that muscle growth induced by strength training is hindered when the testosterone production was inhibited by a GnRH analog (Kvorning et al., 2006). Therefore, our present findings are well in line with previous studies, but it should be noted that the pooled mean of muscle mass difference in the present analysis was close to 0. This actually indicates that in the control group no major declines in muscle mass occurred, making it difficult to detect significant difference between the groups.

Of note is that in the majority of studies (Galvão et al., 2010, 2014; Nilsen et al., 2015; Winters-Stone et al., 2015; Wall et al., 2017; Taaffe et al., 2018), patients had already been treated with ADT for more than 6 months prior to the exercise intervention. A recent study by Taaffe et al. (2019) showed that lean mass was preserved when strength training and concomitant supplementation of calcium and vitamin D was initiated at the same time as ADT, whereas the same training was no longer effective when it was commenced after 6 months. Moreover, during the first 6 months of ADT, significant reductions in lean mass were observed in the no-training control group, highlighting the importance of the timing of physical exercise. This finding was in line with the study by Cormie et al. (2015a), in which lean mass was preserved when combined aerobic and strength training was initiated concomitantly with ADT. In addition, it should be noted that the duration of training interventions in the studies included in this review differed from 3 to 12 months. However, due to the low heterogeneity calculated by our fixed model, a meta-regression was not deemed necessary. Therefore, the small differences in effect sizes between the studies indicate that the duration of the training intervention beyond 3 months does not seem to have a major effect on changes in lean mass.

In addition to the timing of the training, other variables such as the type (Stewart et al., 2014), intensity, (Fyfe et al., 2014), and frequency (Kemmler and von Stengel, 2013) of exercise training need to be considered. Strength training protocols used in the eligible studies included in our meta-analysis mainly consisted of 6–12 repetition maximum. In fact, such a training regimen is well in line with exercise guidelines for healthy populations, which typically recommend intensity ranges from 65 to 85% of 1 RM to optimize muscle hypertrophy (American College of Sports Medicine, 2009; Lasevicius et al., 2018). Similarly, the training frequency (i.e., 2–3 weekly sessions) applied in the majority of the included studies was in agreement with previous studies in healthy populations (Sayers and Gibson, 2012; Filho et al., 2013; Turpela et al., 2017). Therefore, the training characteristics do not seem to aid explanation that the present findings found no significant changes in muscle morphology.

Interestingly, the majority of eligible studies used a combination of aerobic and strength training (Galvão et al., 2010, 2014; Nilsen et al., 2015; Wall et al., 2017; Taaffe et al., 2018). Over the past decades, it has been extensively discussed whether performing aerobic and resistance training within the same exercise program may hinder hypertrophic adaptations (Wilson et al., 2012). This was thought to be mainly due to an inhibition of the mammalian target of rapamycin (mTOR) signaling pathway through AMP-activated protein kinase (AMPK), activated by endurance exercise (Hawley, 2009). For healthy populations, this hypothesis has recently been challenged with studies actually indicating rather additive effects on lean mass when aerobic and strength training are combined (Harber et al., 1985; Konopka et al., 2010; Murach and Bagley, 2016). This is especially in light of the rather low volume of aerobic training performed in the included studies (2–3 times per week, 20–30 min of continues or interval bouts at 70–85% of maximal heart rate) which is unlikely to have a negative impact on morphological adaptations (Hawley, 2009). However, investigating neuromuscular interference was beyond the scope of the present meta-analysis. Future studies may investigate potentially negative adaptations by comparing combined training with strength training only in prostate cancer patients receiving ADT.

When interpreting the present data, one should bear in mind that we purposefully excluded studies in which the intervention included nutritional supplementation, to elucidate the sole effects of exercise training on muscle mass and muscle strength. It is well-known that the synergistic effects of combined training and protein supplementation are much greater than the exercise training stimulus alone for muscle hypotrophy (Tang and Phillips, 2009). It has been shown that ADT reduces basal and protein feeding–induced rises in muscle protein synthesis, whereas concomitant exercise and protein supplementation may reverse these effects (Hanson et al., 2017; Dawson et al., 2018). Thus, even though testosterone plays a major role in maintaining muscle mass, it may not be required for robust responses of muscle protein synthesis following exercise (Hanson et al., 2017).

Despite showing no significant changes in muscle hypertrophy, our meta-analysis indicated positive changes in both lower- and upper-body maximal strength, as has been shown in a previous review, including prostate cancer patients undergoing a variety of treatments (Bourke et al., 2016). Typically, changes in muscle strength are associated with morphological changes, but increases in muscle strength may also occur due to neural improvements, such as an enhanced central motor drive, increased motor neuron excitability, and reduced presynaptic inhibition (Moritani and deVries, 1979; Ahtiainen et al., 2003). Although this aids in explaining our present findings, it should be noted that out of seven studies included in this review, muscle strength was assessed in only five studies, including a total of 331 patients. Thus, further studies are warranted to confirm the associations of muscle hypertrophy and maximal strength in prostate cancer patients undergoing ADT.

There are number of limitations to be considered when interpreting our findings. Unlike previous reviews on a similar field (e.g., Kraemer and Ratamess, 2004; Gardner et al., 2014), we focused on a very homogenous sample of studies and included only RCT's. Controlled-trials without randomization bear a potential risk of bias due to preferential allocation of patients. Moreover, we decided to include only studies in English language, assuming that the peer-review process is more rigorous in renowned international journals. Lastly, the main criteria for inclusion into our analysis was a non-training control group. However, actually only in three studies (Galvão et al., 2010; Cormie et al., 2015a; Nilsen et al., 2015) a true control group with usual care was used, while in the remaining studies placebo-control were implemented (i.e., providing educational material, stretching etc.).

## Conclusion

Our meta-analysis provided evidence that exercise training alone may be effective for increasing muscle strength but may not be sufficient to affect muscle mass in prostate cancer patients undergoing ADT. To maximize the effects of exercise training on muscle hypertrophy, future studies should focus on combined training and nutritional interventions as well as the timing of exercise in relation to the ADT treatment. Moreover, studies should aim at identifying underlying mechanisms by which prostate cancer patients receiving ADT may still be able to gain lean body mass. This, in turn, will help to optimize training recommendations for this population.

## Data Availability

All datasets generated or analyzed for this study are included in the manuscript and/or the supplementary files.

## Author Contributions

ZC, SC, and MS contributed conception and design of the study. ZC and YZ searched the databases. ZC performed the statistical analysis and wrote the first draft of the manuscript. MS, SC, CL and HZ modified sections of the manuscript. All authors contributed to manuscript revision, read, and approved the submitted version.

### Conflict of Interest Statement

The authors declare that the research was conducted in the absence of any commercial or financial relationships that could be construed as a potential conflict of interest.
