# Corrigendum: Supervised Physical Training Enhances Muscle Strength but Not Muscle Mass in Prostate Cancer Patients Undergoing Androgen Deprivation Therapy: A Systematic Review and Meta-Analysis

**DOI:** 10.3389/fphys.2019.01126

**Published:** 2019-08-28

**Authors:** Ziyuan Chen, Yuan Zhang, Chunyan Lu, Hao Zeng, Moritz Schumann, Sulin Cheng

**Affiliations:** ^1^Department of Physical Education, Exercise, Health and Technology Centre, Shanghai Jiao Tong University, Shanghai, China; ^2^The Key Laboratory of Systems Biomedicine, Ministry of Education, and the Exercise Translational Medicine Centre, Shanghai Center for Systems Biomedicine, Shanghai Jiao Tong University, Shanghai, China; ^3^Department of Endocrinology, West China Hospital, Sichuan University, Chengdu, China; ^4^Department of Urology, West China Hospital, Sichuan University, Chengdu, China; ^5^Department of Molecular and Cellular Sport Medicine, Institute of Cardiovascular Research and Sport Medicine, German Sport University, Cologne, Germany; ^6^Faculty of Sport and Health Sciences, University of Jyväskylä, Jyväskylä, Finland

**Keywords:** ADT, androgen suppression, lean mass, exercise medicine, strength training, exercise oncology

In the original article, there was a mistake in Figure 2. In the study of Winters-Stone et al. (2015), the risk of detection bias was mistakenly presented as “uncertain,” which should correctly be shown as “low.” The corrected Figure 2 appears below. This correction does not change the scientific conclusions of the article. The original article has been updated.

**Figure 2:**
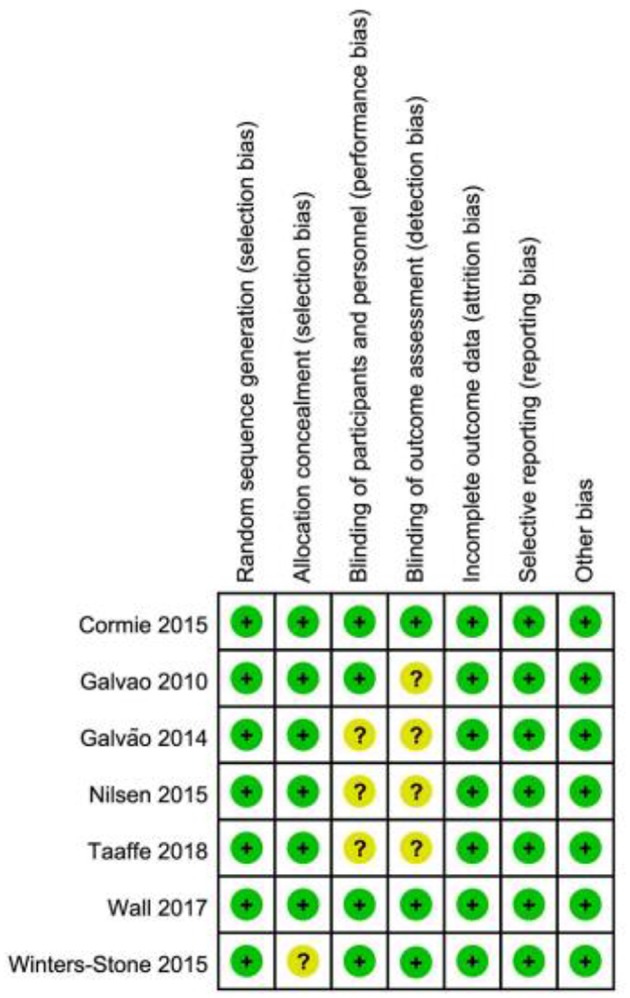
Summary of risk of bias assessment.
